# Filamentous virions act as non-infectious interfering particles to modulate papillomavirus infection

**DOI:** 10.1128/jvi.02039-25

**Published:** 2026-01-13

**Authors:** Dominik van Bodegraven, Sreedeepa Saha, Lilo Greune, Reinhard Kirnbauer, Petra Dersch, Mario Schelhaas

**Affiliations:** 1 University of Münster, Institute of Cellular Virology, ZMBE 9185, Münster, Germany; 2 DFG Research Group ‘ViroCarb: Glycans controlling non-enveloped virus infections’ (FOR2327), Coordinating University of Tübingen, Tübingen, Germany; 3 University of Münster, Institute of Infectiology, ZMBE 9185, Münster, Germany; 4 Laboratory of Viral Oncology, Department of Dermatology, Medical University of Vienna 27271, Vienna, Austria; University of Toronto, Toronto, Ontario, Canada

**Keywords:** virus entry, human papillomavirus, virus structure, virus-host interaction, defective interfering particles

## Abstract

Genus beta (β) human papillomaviruses (HPVs) potentially contribute to the development of non-melanoma skin cancer. Yet, comparatively little is known about their biology. In particular, details about initial infection, i.e., host cell entry, remain mostly elusive. During initial characterization of β HPV5 pseudovirion (PsV) preparations, surprisingly large amounts of filamentous particles were found besides the prototypical icosahedral (T = 7) virions. Whether these filamentous particles actively contribute to or interfere with infectivity of the spherical viruses is unknown. Using a combination of morphological, biochemical, and virological methods, we showed that the filamentous particles are non-infectious. Moreover, they interfered with the initial step of infection, i.e., binding to cellular heparan sulfate proteoglycans (HSPGs), and served as a decoy for soluble glycosaminoglycans, thereby modulating infectivity by enhancing infectious PsV binding. This explains previous seemingly contradictory findings on HPV5 binding to HSPGs. Importantly, in HPV5 skin warts from an immunocompromised patient, no filamentous particles were observable highlighting differences in the assembly of pseudovirions and native viruses.

**IMPORTANCE:**

Papillomaviruses contribute to numerous cancer incidents and significant mortality despite available vaccinations. Hence, high-risk α HPVs have been the focus of most research in the past. However, there are indications that less well-studied β HPVs may also contribute to certain malignancies. Little is known about their mode of cell invasion, and available data appear partially contradictory. Our work demonstrated that HPV5 as a model β HPVs yielded high amounts of non-infectious filamentous particles during PsV production. These acted as modulators of infection by the infectious spherical particles. Removing these filamentous particles showed that HPV5 engaged HSPGs as the primary receptor for cell binding, similar to high-risk α HPV, indicating a conserved feature not only among α, but also among β HPVs, thereby explaining previous contradictions.

## INTRODUCTION

Human papillomaviruses (HPVs) are small, non-enveloped DNA viruses that infect squamous epithelia of skin or mucosa. The 16 high-risk types of HPVs are known as causative agents for a variety of anogenital and oropharyngeal malignancies, most prominently cervical cancer (1). With about 630,000 new annual cases of HPV-related cancers worldwide, HPVs are considered a major health burden (2).

The double-stranded circular DNA genome of papillomaviruses is packaged into icosahedral (T = 7) particles built by the major and minor capsid proteins L1 and L2, respectively (3–5). L1 self-assembles into pentameric capsomers and capsids that consist of 72 capsomers (3, 6). About 12–72 copies of L2 are located mostly within the capsid lumen, inside the internal cavity of the capsomers (7). Interpentameric disulfide bonds between L1 proteins stabilize the capsid upon maturation (8–10).

Based on the similarity of the L1 gene, the PV types are grouped into different genera, five of which (termed α, β, γ, μ, ν) infect humans (11–13). HPVs of the α genus predominantly display a tropism for mucosal tissue and comprise all high-risk types (14–16). In contrast, β and γ HPVs regularly infect the skin. While it is generally thought that the tissue tropism is rather strict for individual types, this concept has recently been challenged, as particular HPV types are found more often in both mucosa and skin (reviewed in reference 17). While ubiquitous and generally benign, infections of the β HPVs are less well understood than those of the α types (18–20). Importantly, their contribution to non-melanoma skin cancer upon UV-dependent DNA damage is being discussed (21, 22). The β HPV types 5 and 8 are classified as potentially carcinogenic, as they exhibit a strong association with epidermodysplasia verruciformis, a rare, hereditary disease that often leads to squamous cell carcinomas of the skin in sun-exposed areas (14, 23).

The viral life cycle of HPVs is tightly linked to keratinocyte differentiation (24, 25). HPVs initially infect mostly undifferentiated basal stem cells of squamous epithelia and establish a so-called maintenance replication resulting in limited copy numbers of the viral genome (26, 27). Progression of the viral life cycle depends on the differentiation into suprabasal, spinous cells, promoting amplification replication (28–31). Terminal differentiation of keratinocytes into granular cells reduces genome amplification, and elevated expression of the structural proteins L1 and L2 facilitates assembly of new virus particles (32, 33). This complex life cycle complicates the cultivation of viruses *in vitro*. As a consequence, the preparation of pseudoviruses (PsVs) has been developed as a surrogate system for studies of initial infections (34, 35). The PsVs are virus-like particles (VLPs) that harbor reporter gene-expressing pseudogenomes. Expression of the reporter thereby indicates a successful initial infection.

Virus entry is the first important step during the viral life cycle. It comprises all steps that deliver the viral genome to the sites of transcription and replication, from cell binding to nuclear delivery. For HPV16 and other α HPV, entry begins with L1-mediated binding to the glycosaminoglycan of heparan sulfate proteoglycans (HSPGs) (36–39). Four different L1 peptides have been suggested as interaction sites for heparan sulfates (HS) (40, 41). In addition to primary attachment, the interaction with HSPGs stabilizes a particular capsid conformation of virions essential for infection, a feature that was termed structural activation (42–44). Binding and structural activation may occur in a sequential manner (41). Structural activation facilitates proteolytic cleavage of L1 by the secreted, cellular protease kallikrein-8 (45). This, in turn, allows externalization of the L2 N-terminus from the capsid lumen assisted by cellular cyclophilins (46, 47). Subsequent cleavage of the L2 N-terminus by furin, another secreted cellular protease, decreases the affinity for HSPGs and triggers transfer of the virus to an elusive secondary receptor (complex) (47–51). Secondary receptor candidates include integrin α6, growth factor receptors, the annexin A2 heterotetramer, and the tetraspanins CD63 and CD151 (52). Receptor switching likely triggers uptake into cells by a novel endocytic mechanism termed WASH-mediated endocytosis (53–55). Intracellular trafficking through the endosomal system and retrograde transport leads the virus to the Golgi apparatus (53, 56, 57). As the last step in the entry program, nuclear import of the viral DNA occurs after nuclear envelope breakdown during mitosis by tethering to mitotic chromatin (58–60).

Thus, a general outline of HPV entry exists, while many mechanistic details remain elusive. Conceivably, HPVs from other genera may follow the same itinerary. In support, β HPVs require furin-mediated L2 cleavage (61), retromer function (57), an active ESCRT machinery (62), γ-secretase (61, 63) for intracellular trafficking, and tethering to mitotic chromatin for nuclear import (58), all of which is similar to α HPVs. While these findings are mostly consistent with a certain degree of conservation for entry of all HPVs, a systematic comparison between α and β types is lacking.

Notably, certain observations for β HPV entry are partially inconsistent. For example, the use of HSPGs as a primary receptor is under debate for the β HPV5. In contrast to α HPVs, heparin as an HS analog insufficiently blocks HPV5 infection, which has been interpreted as an HS-independent cell binding (38, 64). In line with this notion, *in silico* surface charge modeling of HPVs indicated positive versus negative net surface charges of α versus β/γ type virions, respectively (65). A negatively charged surface of β HPVs might thus impair interaction with negatively charged HS. In contrast, other work indicated that HPV5 can engage heparin during affinity chromatography, and that HS removal impairs infection (38). Thus, it remains unclear if and how the HS-HPV5 interaction impacts infection, and by extension, whether binding and structural activation of α and β HPVs are conserved.

In preparation for a detailed characterization of β HPV entry in comparison to α HPV16, we observed that low infectivity of HPV5 PsV preparations correlated with a high abundance of non-infectious filamentous particles. Using the example of HS binding, we demonstrated that these filamentous particles functioned as non-infectious interfering particles by acting as high-avidity decoys for HS and heparin, and that they modulated the facultative engagement of spherical HPV5 to HS, thereby explaining previous inconsistencies. Importantly, filamentous HPV5 production could not be observed in patient warts, suggesting that it is a feature of the PsV production. Based on a small survey of PsV preparations of various HPV types, filamentous, non-infectious interfering particle formation is favored for β HPVs during PsV assembly, highlighting potential differences in assembly kinetics between α and β types.

## RESULTS

### Characterization of HPV5 pseudoviruses

**Fig 1:**
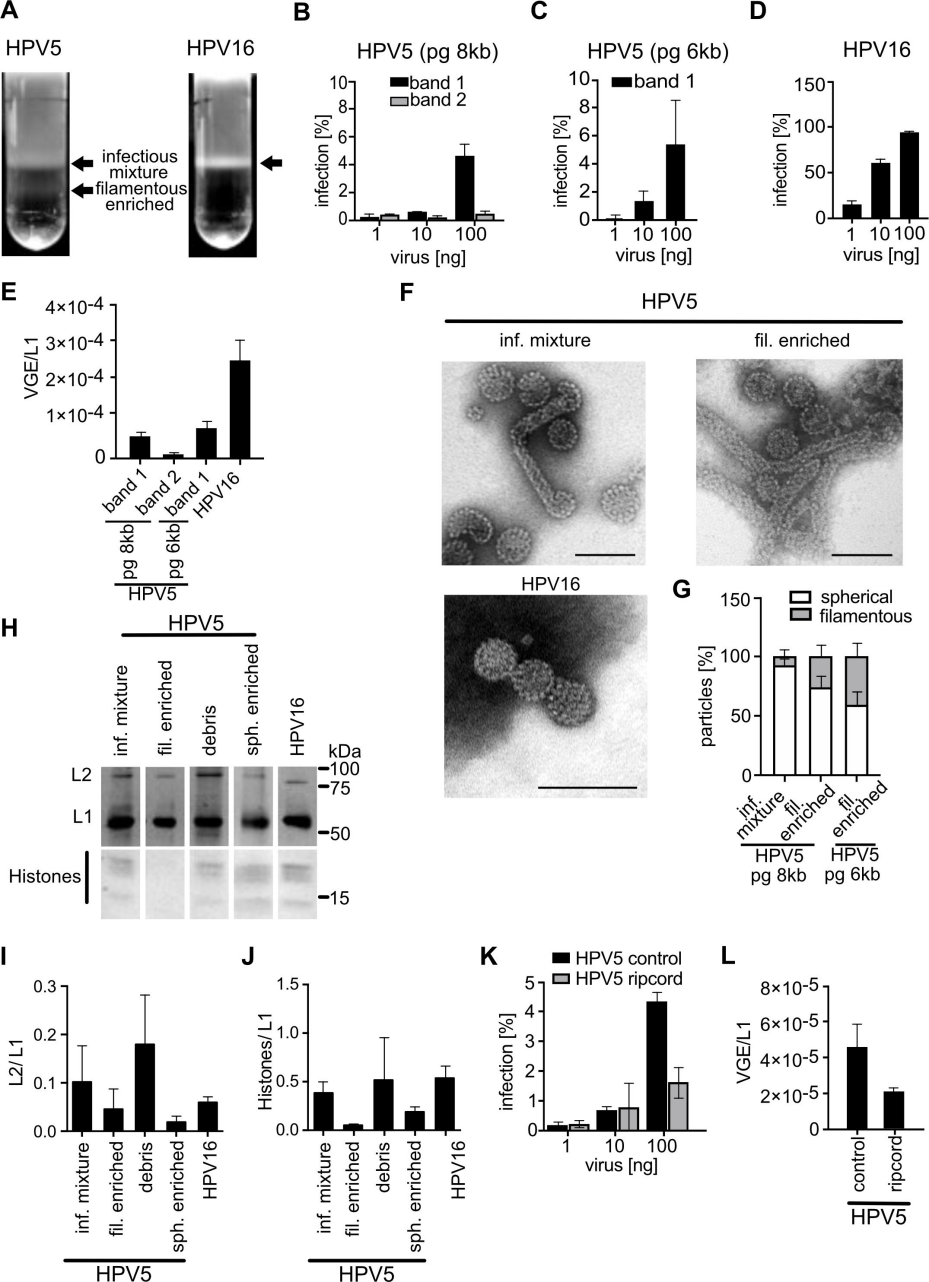
Characterization of HPV5 PsVs. (**A**) Banding pattern (indicated by black arrows) of HPV16 and HPV5 after rate-zonal centrifugation on a continuous OptiPrep gradient (25–39%). (**B**) HeLa cells were infected with the HPV5 PsV fractions at indicated amounts for 48 h. GFP-positive cells (infected cells) were measured by flow cytometry and depicted as average in % of total cells for three independent experiments ± standard deviation (SD). Note the absence of infection for filamentous-enriched HPV5 particles with p8fwB pseudogenome (pg 8 kB). (**C**) As in panel **B**, using HPV5 band 1 particles with the pClneo pseudogenome (pg 6 kB). (**D**) As in panel **B**, using HPV16 PsV. (**E**) The number of viral genome equivalents (VGE) was obtained by qPCR using a standard of viral pseudogenomes and related to the amount of L1 molecules. Depicted is the average of three independent experiments ± SD. (**F**) Representative electron micrographs of negatively stained HPV preparations/bands as indicated. Scale bars are 100 nm. (**G**) Quantification of the percentage of spherical and filamentous particles from panel **F** and filamentous-enriched band of HPV5 pClneo prep (image not shown). Spherical and filamentous particles of HPV5 were counted manually. Values are given as the average of three independent experiments ± SD. (**H**) Coomassie-stained SDS-PAGE gel of different HPV bands obtained from rate-zonal centrifugation. Indicated are the major and minor capsid proteins L1 and L2, as well as cellular histones from the chromatinized pseudogenomes. (**I, J**) Densitometric quantification as shown in panel **H**. Displayed are the averages of (**H**) L2 or (**I**) histone signals relative to L1 signals from three independent experiments ± SD. (**K**) HeLa cells were infected with HPV5 pg 8 kb infectious mixture prepared by the classical method (control) or ripcord method for 48 h. GFP-positive (infected) cells were measured by flow cytometry and depicted as % of total cells for three independent experiments ± standard deviation (SD). Ripcord method decreases infection rates instead of improving infectivity. (**L**) VGE obtained by qPCR of the PsV preps is shown in panel **K**.

Initially, we set out to characterize HPV5 PsV preparations. While linear velocity gradient-based purification of HPV16 PsVs (35) typically resulted in one homogeneous band of virus particles, HPV5 purifications consistently exhibited two distinct virus populations (Fig. 1A). Of these, only the upper, lighter fraction comprised infectious particles and is here referred to as infectious HPV5 mixture. In line with previous observations (66), the infectious HPV5 mixture infected HeLa cells much less efficiently than HPV16 preparations (Fig. 1B through D). This phenotype was independent of the size of the pseudogenome (pg) used in the preparation (Fig. 1B and C), despite the fact that using smaller pgs typically improves HPV16 infectivity by increasing pg incorporation rate during PsV production (34). To test whether the infectious HPV5 mixture harbored significantly more non-infectious particles than HPV16 to result in lower infectivity, we assessed the amount of viral genome equivalents in a preparation, i.e., the relative number of pg-containing particles, by quantitative polymerase chain reaction (qPCR). Relative to the L1 content, HPV5 contained about 4.5-fold fewer VGE than HPV16 preparations (Fig. 1E), although, notably, HPV5 infectivity was reduced by about 60-fold compared to HPV16 (compare Fig. 1B and D). Thus, the reduced amount of VGE accounted only for a small part of reduced infectivity (compare Fig. 1B through D). For certain HPV types, the infectivity/particle ratio can be improved by reducing the amount of particles that incorporate genomic DNA from producer cells by using the so-called “ripcord” preparation method (67). For HPV5, this was not the case, both in terms of infection (Fig. 1K) and VGE/particle ratio (Fig. 1L). Furthermore, we tested whether incorporation of the minor capsid protein L2, an important mediator of HPV entry, or of histones was affected in HPV5. Based on the quantification of band intensity after SDS-PAGE and Coomassie staining, HPV5 L2 and histone contents of the infectious HPV5 mixture were comparable to HPV16, indicating that they were incorporated efficiently and not causative for reduced HPV5 infectivity (Fig. 1H through J). Unsurprisingly, the non-infectious fraction of HPV5 (termed “filamentous-enriched HPV5” for reasons that will become apparent in the next paragraph) exhibited no detectable pg incorporation and reduced the amounts of L2 and histones per L1 (Fig. 1H through J).

Since the composition of HPV5 virions failed for the most part to explain the low infectivity compared to HPV16, the structural integrity of viral particles was assessed next by negative-staining electron microscopy (EM). Both HPV5 and HPV16 preparations similarly contained intact spherical particles of 55 to 60 nm in diameter, without noticeable differences in particle morphology (Fig. 1F). Strikingly, both HPV5 populations contained significant amounts of filamentous particles that were absent in HPV16 preparations (Fig. 1F and G). The number of filamentous particles was highest in the non-infectious fraction, where the filamentous almost equaled spherical particle numbers for HPV5 particles produced with a 6kB pg (Fig. 1G). Hence, we designated this fraction as “filamentous-enriched HPV5,” as mentioned above. The filamentous HPV5 particles displayed a rather invariable width of about 30 nm, whereas the length exhibited a broad dispersity ranging from about 200 nm up to several microns.

Since the virus particles of the filamentous-enriched HPV5 fraction were devoid of pg and displayed decreased L2/L1 and histone/L1 ratios, it is likely that filamentous particles are *per se* non-infectious. What if filamentous HPV5 particles acted as defective interfering particles as observed for other viruses, such as dengue virus and influenza A virus (IAV) (68–71)? Then, the presence of filamentous particles in the infectious HPV5 mixture would account for its lower HPV5 infectivity compared to HPV16, which lacks filamentous particles.

### Enrichment of spherical particles increases infection

**Fig 2:**
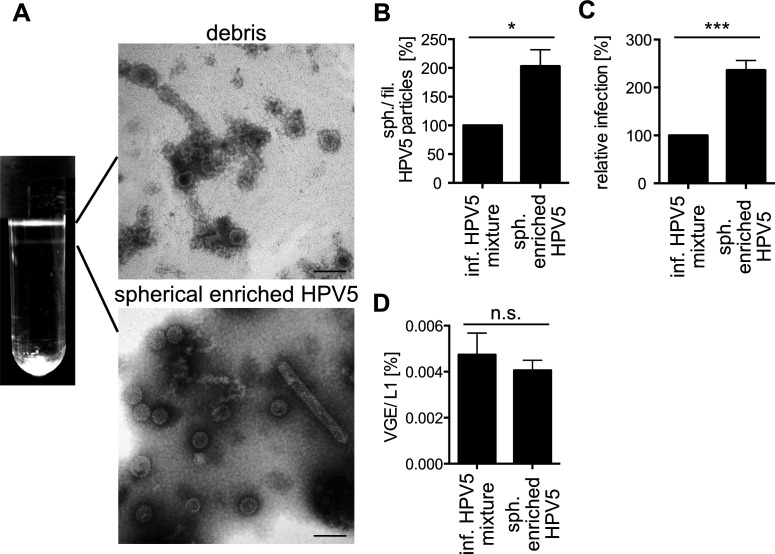
Enrichment of spherical particles increases infection. (**A**) Banding pattern of infectious HPV5 mixture after centrifugation on a step gradient containing 50, 90, 95, and 100% saturated KBr cushions. Connected by lines are representative electron micrographs of the respective particle population. Scale bars indicate 100 nm. (**B**) Quantification of EM images in panel **A**. Spherical and filamentous particles of the spherically enriched HPV5 fraction were counted manually and depicted as spherical/filamentous particle percentage relative to the infectious HPV5 mixture. Values are given as the average of three independent experiments ± SD. (**C**) HeLa cells were infected for 48 h, and infection was scored by flow cytometry as the average of GFP-positive cells in % of total cells relative to the infectious HPV5 mixture for three independent experiments ± SD. (**D**) VGE were determined by qPCR using a pseudogenome standard. The number of VGE is displayed relative to the number of L1 molecules as the average of three independent experiments ± SD. Statistical significance was tested by unpaired *t*-test using GraphPad Prism v6 with *P* values: * *P* < 0.05, ** *P* < 0.01, *** *P* < 0.001 or insignificant (n.s.).

To address this question, the ratio of spherical vs filamentous particles of the infectious HPV5 mixture would need to be improved, or the spherical particles separated from the filamentous particles. Since our attempts to fully separate spherical and filamentous particles by size exclusion chromatography or various rate-zonal centrifugation approaches with continuous gradients were unsuccessful, we followed the approach of Mattern and colleagues (72). Step-gradient-based velocity ultracentrifugation using different potassium bromide cushions yielded the separation of two bands: the first of which exhibited mostly particle debris (HPV5 debris), whereas the second contained mostly spherical particles with clearly reduced numbers of filamentous particles (referred to as spherically enriched HPV5) (Fig. 2A and B). Spherically enriched HPV5 exhibited more than two-fold increase of spherical particles, which correlated with an increase of infectivity to a similar extent (Fig. 2C). Since the amount of reporter plasmid per L1 molecule remained unchanged (Fig. 2D), the increase in infectivity thus correlated with the removal of filamentous particles, indicating that filamentous HPV5 impaired the infectivity of spherical HPV5.

### HPV5 binds to HS for infection

**Fig 3:**
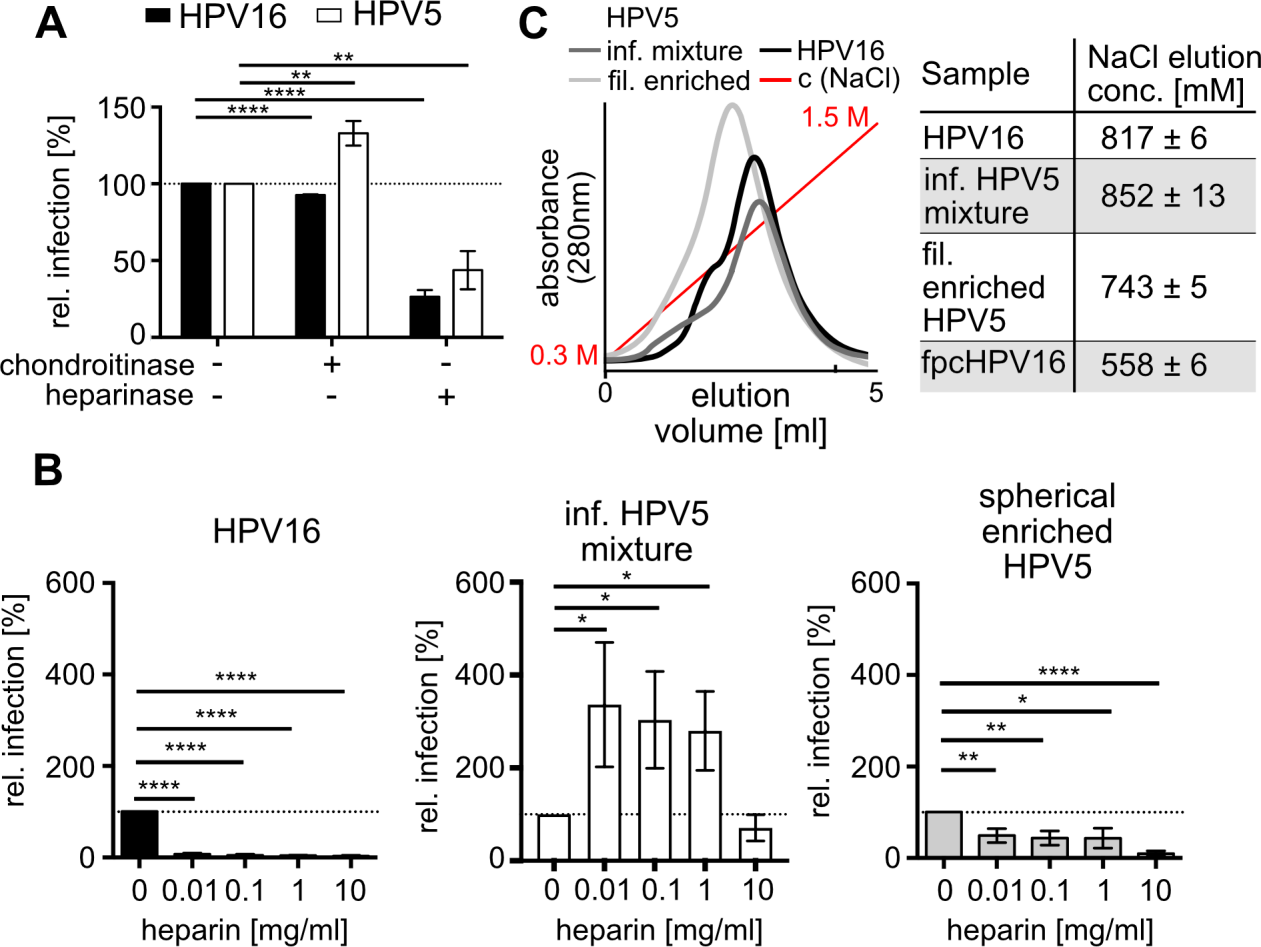
HPV5 binds to HS for infection. (**A**) Cells were treated with heparinase or chondroitinase for 1 h prior to infection with HPV16 or HPV5 (infectious mixture). Infection was scored 48 h p.i. by flow cytometry. Depicted is the average of infected cells in % relative to the untreated control for three independent experiments ± SD. (**B**) HPV16 or the different HPV5 fractions were preincubated with the indicated concentrations of heparin for 1 h prior to infection. Infection was scored, and results are displayed as in panel **A** relative to the heparin-untreated samples. (**C**) Heparin affinity chromatography of HPV16 or different HPV5 fractions. Displayed is a representative elution profile in relation to the increasing concentration of NaCl as eluant (left). The peak of the elution profile was related to the NaCl concentration, which is depicted as average concentration values of three independent experiments ± SD. The elution concentration of furin precleaved HPV16 was obtained in (48) and is shown as a reference for significant changes. Statistical significance was tested by unpaired *t*-test using GraphPad Prism v6 with *P* values: *P < 0.05, **P < 0.01, ***P < 0.001, ****P < 0.0001 or insignificant (not indicated).

Our results so far suggested that filamentous particles acted as defective interfering particles. To substantiate this, we addressed how filamentous particles would mechanistically interfere with entry. The first step of virus entry is attachment to cells. Perhaps filamentous particles competitively interfered with binding more efficiently than non-infectious spherical particles due to their structural differences. As HPV5 binding to cellular HSPGs is still controversial, we first confirmed the importance of sulfated glycosaminoglycans for binding on the cell surface. For this, cell surface HS or chondroitin sulfates (CS) were removed by treating cells with either heparinase or chondroitinase, and treated cells were subsequently infected. In line with previous work and similar to HPV16 (38), CS removal impaired HPV5 infectivity only to a minor extent, whereas HS removal strongly reduced HPV5 infection (Fig. 3A). This indicated the importance of HS on the cell surface. In contrast to HPV16, inhibitory competition for HSPG binding with heparin, a soluble HS analog, requires much higher concentration for HPV5, is inefficient even at high concentrations, and remarkably leads to more efficient infection at low concentrations in stark contrast to HPV16 (38, 61). Challenging the infectious HPV5 mixture with heparin replicated these results as expected (Fig. 3B). In contrast, spherically enriched HPV5 preparations with reduced amounts of filamentous particles exhibited a dose-dependent decrease in infection upon heparin challenge (Fig. 3B). While this effect was consistent in all spherically enriched HPV5 preparations, the degree was somewhat variable, likely as a result of how well spherical particles were enriched. This suggested that the non-infectious filamentous particles somehow interfered with heparin’s ability to impair infection of infectious spherical particles. Of note, spherically enriched HPV5 was still inhibited less efficiently than HPV16. This might be perhaps explained by the remaining filamentous particles. However, this seemed less likely, since about 100-fold more HPV5 than HPV16 PsVs were needed to achieve similar numbers of infected cells. Thus, we presume that a high abundance of non-infectious spherical particles in HPV5 preparations is likely, which in turn contributes to a lower efficacy of heparin-mediated attachment inhibition.

In summary, the presence of filamentous particles in the infectious HPV5 mixture allowed infection despite the presence of heparin and, at low heparin doses, even increased infectivity. A possible explanation for this would be that the filamentous particles engaged sulfated glycosaminoglycans more efficiently, and thereby, heparin could no longer compete with the binding of the infectious spherical HPV5 particles to cells. This would be achieved by higher affinity and/or avidity of filamentous HPV5 toward HS and heparin.

To test whether a higher affinity plays a role, the infectious HPV5 mixture and filamentous-enriched HPV5 fraction were subjected to heparin affinity chromatography. The concentration of sodium chloride needed to release the particles from the column correlates with charge-based affinity toward heparin (48). For elution, both HPV5 populations demanded sodium chloride concentrations similar to HPV16, indicating similar affinities of the different HPV5 preparations and HPV16 toward heparin (Fig. 3C). From this, we inferred that the differing avidities of filamentous versus spherical particles likely account for the differences in engaging heparin.

Overall, it is thus likely that filamentous HPV5 acted as a high-avidity decoy for engagement of HS and heparin, providing the first insights into their potential role as defective interfering particles. Moreover, this data also strongly indicated that HPV5 binding to cells occurs through HS binding. The combined data thus provided a rationale for the different conclusions of previous work with regard to HS binding (38, 64, 65).

### Non-infectious particles act as decoys for heparin and heparan sulfate proteoglycans

**Fig 4:**
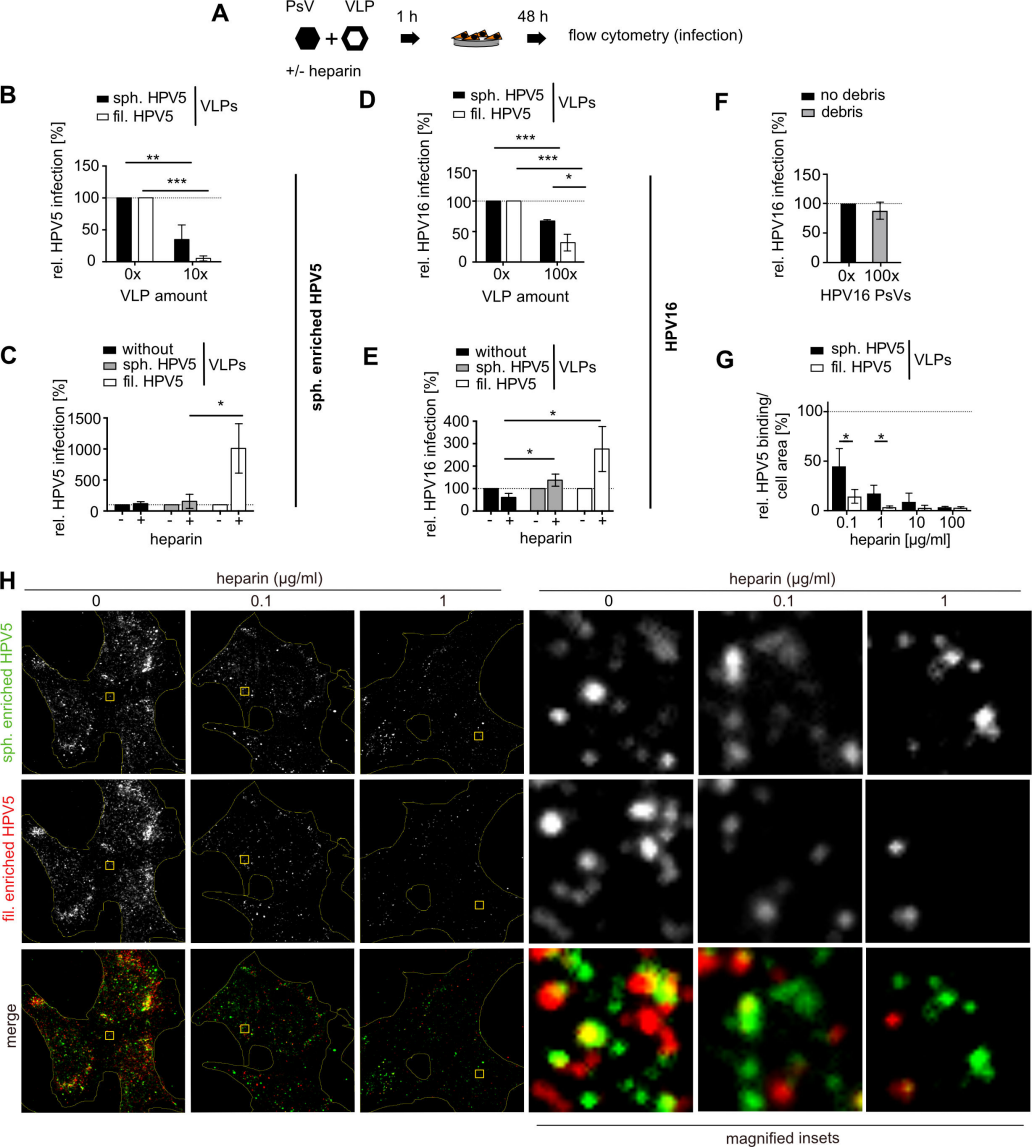
VLPs as decoys for heparin and cell surface HSPGs. (**A**) Schematic depiction of the assay, where HPV PsVs and VLPs were mixed and preincubated with or without heparin for 1 h prior to infection of HeLa cells. After 48 h, infection was scored as in Fig. 3A. (**B, C**) 100 ng spherically enriched HPV5 PsVs were mixed with 10× molarity of capsomers of either filamentous- or spherically enriched HPV5 VLPs as indicated and incubated without (**B**) or with (**C**) 10 µg/mL heparin prior to infection. Striated lines indicate infectivity without VLPs throughout the figure. (**D–E**) As shown in panels **B and C,** using 5 ng HPV16 PsVs and filamentous- or spherically enriched HPV5 VLPs. After preincubation without (**D**) or with (**E**) 1.6 µg/mL heparin, HeLa cells are infected. In panel **E,** 100× VLPs compared to PsVs are used. Due to their higher infectivity, significantly less HPV16 was required to achieve infection levels comparable to HPV5. To compare similar molar heparin/L1 ratios, less heparin was used in panels D and E compared to the HPV5 experiments. (**F**) HPV16 PsVs were mixed with 100× L1.1 debris, obtained in Fig. 2A, and added to HeLa cells for infection. (**G, H**) Fluorescently labeled spherically or filamentous-enriched HPV5 fractions were mixed with equimolar L1 content to attain 100 ng L1 in total, preincubated with the indicated heparin concentrations for 1 h, and subsequently added to cells. After 1 h of binding, cells were subjected to confocal microscopy. (**G**) Quantification of virus spots per cell area of at least 10 fields of view per sample and experiment was determined computationally and normalized to the heparin-untreated sample. They are displayed as averages of three independent experiments ± SD. Statistical significance was tested by an unpaired *t*-test using GraphPad Prism v6. Obtained *P* values are displayed with asterisks: * *P* < 0.05, ** *P* < 0.01, *** *P* < 0.001 or insignificant (not indicated). (**H**) Depicted are representative images as sum projections of confocal stacks. Cell outlines as determined by phalloidin staining are indicated by yellow lines.

To challenge the emerging model, in which filamentous HPV5 particles acted as a high-avidity decoy for HS or heparin, we tested whether titrating in filamentous HPV5 VLPs would indeed confer higher competitive effects than spherical HPV5 VLPs on PsV infectivity. For this, L1+L2 VLPs, i.e., particles without pg, were added in increasing amounts to PsVs of either spherically enriched HPV5 or HPV16, and infectivity was tested with or without heparin challenge (Fig. 4A). To ensure a similar number of HS-engaging sites of spherical and filamentous VLPs for accurate comparisons, the same molarity of L1 capsomers of spherical or filamentous VLPs was added; i.e., fewer filamentous VLPs were added than spherical VLPs. Notably, addition of filamentous HPV5 VLPs much more prominently reduced infection of spherically enriched HPV5 PsVs than addition of the same molarity of spherically enriched HPV5 VLPs (Fig. 4B), indicative of a better binding to cells via HSPGs. If infections were carried out with the addition of heparin as a binding inhibitor in the presence of VLPs, only the presence of filamentous-enriched HPV5 led to significantly increased infectivity (Fig. 4C). This is in line with a preferred binding of filamentous particles to heparin, which would result in less inhibition of binding of the infectious spherical particles to cells. These results strengthen the notion that filamentous HPV5 particles are more competent binders for both HSPGs and heparin than spherical particles.

These results were replicated when spherical or filamentous HPV5 VLPs were added to HPV16 PsVs with filamentous HPV5 VLPs, exhibiting a higher potency to interfere with HPV16 infection (Fig. 4D). In the case of heparin preincubation, adding VLPs reduced the efficacy of heparin to inhibit HPV16 infection (Fig. 4E). Again, filamentous VLPs exhibited more pronounced effects than spherical VLPs, supporting that filamentous VLPs more efficiently acted as a decoy for heparin engagement (Fig. 4E). To corroborate that the number of potential binding sites per particle or L1 structure was important, the HPV5 debris fraction mostly containing subviral structures and L1 pentamers (Fig. 2A) was employed as binding competitor for HPV16 PsVs (Fig. 4F). No significant reduction of infection was observed, indicating that higher order particles are needed to compete for virus binding due to their higher avidity (compare Fig. 4D and F). In summary, filamentous VLPs competed more efficiently than spherical VLPs and subviral structures with spherical PsV for engagement of HSPGs or heparin, most likely due to higher avidity for binding.

To corroborate a more efficient binding of filamentous over spherical particles to HS, virus binding to cells was analyzed by microscopy similar to the infection experiments. For this, spherically and filamentous-enriched HPV5 were covalently labeled with two distinct fluorophores. Those labeled particles were added to cells, reflecting similar L1 amounts, i.e., less filamentous than spherical particles but a similar amount of capsomers. In the presence of heparin, filamentous HPV5 particles exhibited a stronger decrease in binding to cells, indicating that they engaged heparin more efficiently than the spherical particles (Fig. 4G and H). This confirmed that filamentous HPV particles competed more efficiently with their infectious spherical counterparts for HS binding.

### *In vivo* HPV5 particle assembly

**Fig 5:**
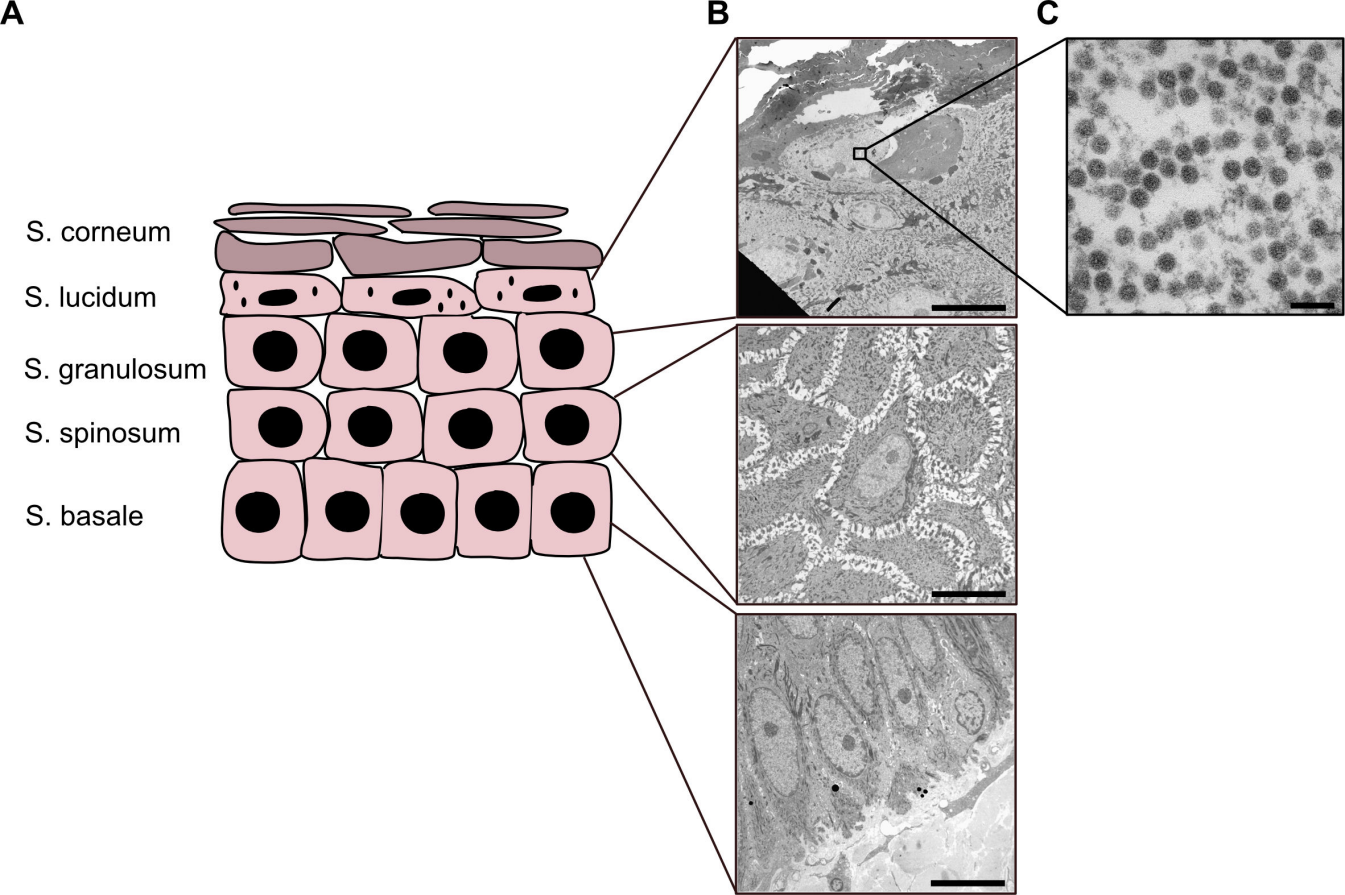
HPV5 particle assembly in a patient wart. (**A**) Representation of the cellular organization in human epidermis tissue: stratum corneum, stratum lucidum, stratum granulosum, stratum spinosum, and stratum basale. (**B**) Representative electron micrographs of the indicated cell layers. Scale bar indicates 10 µm. (**C**) Magnified inset of a cell from stratum granulosum. Note the presence and absence of spherical and filamentous HPV5 virus particles, respectively. Scale bar indicates 100 nm.

Since HPV5 filamentous PsV acted as defective interfering particles for binding *in vitro*, and since cottontail rabbit papillomaviruses (CRPV) have been observed to form filamentous particles (73, 74), the relevance of HPV5 filamentous particles for *in vivo* infections remains in question. As a prerequisite, HPV5 would have to actually form filamentous particles during *in vivo* infection. To investigate this, we obtained HPV5-positive warts from an immunocompromised patient (75). These warts were subjected to thin-section EM, and sections of the different layers of the squamous epithelium (stratum basale to stratum granulosum; Fig. 5A) were analyzed for the presence of assembled HPV5 particles. Assembly of HPVs typically occurs in the stratum granulosum/lucidum, but not the stratum basale or stratum spinosum (32, 76). Hence, HPV5 particle assembly was not observed in the lower layers of the epithelium (Fig. 5B). However, innumerable HPV5 particles were observed in nuclei of cells within the stratum granulosum/lucidum (Fig. 5B and C). Importantly, no filamentous HPV5 particles were observed. While this data from one patient sample does not formally rule out circumstantial formation of filamentous HPV5 particle *in vivo*, it fails to support a role of filamentous HPV5 particles during *in vivo* infection. Rather, the data point to an aberrant assembly of filamentous particles during PsV production.

### β HPVs are more prone to filamentous particle formation

**Fig 6:**
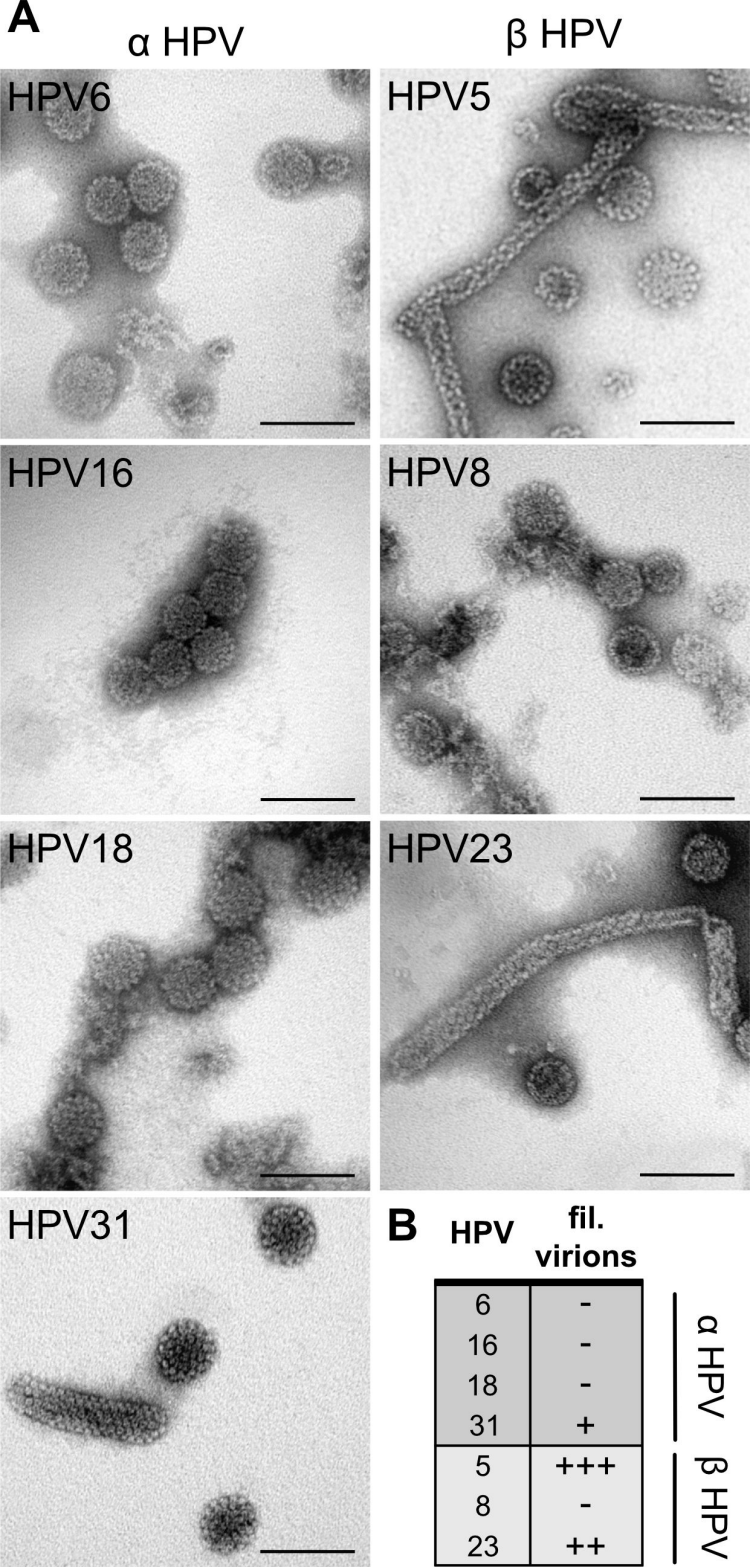
β HPVs are more prone to filamentous particle formation. (**A**) Representative electron micrographs of the indicated HPV PsVs after negative staining. Scale bar corresponds to 100 nm. (**B**) Qualitative assessment of the abundance of filamentous particles in HPV PsV preparations based on (**A**) with – (not detected) to +++ (very high abundance).

Finally, we wondered whether the potentially aberrant formation of filamentous particles during PsV production was restricted to HPV5, as HPV16 preparations did not exhibit any. Thus, we analyzed a set of α and β HPV preparations for the presence and abundance of filamentous particles using negative EM staining. Filamentous particles were detected in samples of α HPV31, β HPV5, and HPV23, whereas α HPV6, HPV16, and HPV18 were devoid of filamentous particles (Fig. 6A). It is noteworthy that the number of filamentous HPV31 particles was not only low, but that these filamentous particles were rather small. Overall, it appeared that β HPV PsV were more prone to form filamentous particles than their α HPV counterparts (Fig. 6B).

## DISCUSSION

This work demonstrated an abundance of filamentous in addition to spherical HPV5 particles in PsV preparations. These non-infectious filamentous particles functioned as defective interfering particles by acting as potent decoys for HS glycosaminoglycans, thereby modulating HPV5 PsV infection *in vitro*. Moreover, based on a limited selection, β HPV types were more prone to form filamentous PsV particles than α HPV types. In support of this, Kwak and colleagues (58) showed that increased infection after heparin challenge predominantly occurs for β types. Thus, our findings have crucial implications for experimental studies of HPV entry.

Our findings on how filamentous particles modulate HPV5 infection affect our understanding of, and approaches to studying, HPV cell entry. Previous work showed that HPV5 infection is insensitive to inhibition by heparin (38, 61, 64, 65). Based on this finding, a facultative use of HSPGs as binding receptors for HPV5—and by extension, as conserved binding receptor among HPVs of different genera—has been called into question. Here, we demonstrated that HPV5 PsV preparations contain a considerable amount of filamentous particles. These acted as high-avidity decoys for heparin and thereby hampered the interpretation of competitive binding studies as previously published. Enrichment of spherical HPV5 particles led to a dose-dependent decrease of infection if challenged by heparin. Together with the requirement for HSPGs and heparin binding (this work, and reference 38), this strongly indicated that engagement of HSPGs for binding is conserved in HPV5 and likely other β HPV. Highly sulfated HS stabilizes a particular conformation of HPV16 virions to support receptor switching and uptake (42–44). To explain the increase of HPV5 infectivity in the presence of heparin, previous work proposed that heparin might introduce an additional conformational change in HPV5 virions to facilitate L2 cleavage by furin (61). Since the presence or absence of filamentous particles reversibly changed the efficacy of heparin inhibition, the existence of additional conformational changes in β HPV5 virions now appears less likely. Enrichment of spherical, infectious HPV5 particles thus provided a better understanding of HPV5 cell entry and may be crucial for studying β HPV and potentially other types.

It is intriguing to note that while HPV5 PsV exhibited the formation of filamentous particles, no evidence for *in vivo* formation was obtainable. This strongly indicates aberrant particle assembly during PsV production. Yet, based on purified particles from warts, CRPV forms filamentous particles *in vivo* (73, 74). Hence, we cannot formally rule out a putative role for filamentous papillomavirus, at least for CRPV, during productive *in vivo* infection, and it remains tempting to speculate on it. Moreover, these observations beg the question of how the formation of spherical vs filamentous particles would be favored.

For any putative role of filamentous particles *in vivo*, we currently have to rely on our *in vitro* results. *In vitro*, the filamentous HPV5 PsV acted as defective interfering particles, which were originally defined as naturally occurring virus particle variants that directly interfere with the intracellular replication of the wild-type virus (69). The filamentous HPV5 PsVs were designated as defective interfering particles because they competed very efficiently for the cell surface receptor, a feature that, to our knowledge, is unique among viruses. Defective interfering particles were detected for various viruses, including IAV (71), dengue virus (68), or vesicular stomatitis virus (VSV) (77). The defective interfering particles of IAV are well investigated and often serve as a paradigm. Their genomes are either truncated (71, 78) or have accumulated point mutations (79). They interfere with viral replication by competing for the replication machinery (80) or for packaging (81), or they stimulate the immune system (80). While the mode of interference of defective interfering particles is well investigated, little is known about their biological relevance. They may reduce viral fitness (68), force the infectious virus population into a periodic growth pattern (82), or facilitate the establishment of persistent infection (83, 84). For papillomaviruses, a reduced viral fitness during transmission is conceivable based on the higher avidity of filamentous particles. A speculative role as an immune decoy, however, can also be envisioned.

While papilloma- and polyomaviruses can form filamentous particles (4, 72), there is little information on the parameters that drive such formation. The apparent difference in HPV5 PsV particle formation vs HPV5 native virion formation in a patient wart potentially provides insights. PsV production involves a strong expression of capsid proteins in packaging cells, driven by strong promoters and L1/L2 sequences optimized for human codon usage (34). In contrast, the L1/L2 expression kinetics *in vivo* are likely lower (85). Capsid protein overexpression may influence the fidelity of capsomer interaction in a HPV type-dependent manner, thereby leading to an erroneous arrangement and, consequently, to the formation of filamentous particles. For T = 7 icosahedral structures, it is known that capsomers with six-fold symmetry build the triangular planes while those with five-fold symmetry are necessary for the curvature (86). An overabundance of capsomers may lead to predominant formation of hexavalently organized capsomers. In fact, simulations of particle assembly show a complex interrelationship between capsomer concentration and particle assembly kinetics/thermodynamics (87). Filamentous particles of viruses, such as ebolavirus, typically comprise helically arranged capsomers in six-fold symmetry (88). Thus, HPV5 particles may fail to assemble in the correct five-fold symmetry upon high expression of capsomers.

In summary, HPV5, as a representative of the β HPV genus, requires HSPGs for cell binding, as do other HPVs. Previously documented insensitivity to heparin can be explained by the presence of non-infectious filamentous particles that acted as decoy to heparin, thereby designating them as a novel type of defective interfering particles. Therefore, it will be important to use infectious, spherically enriched HPV5 particles to further study the mechanism of entry. Finally, how filamentous papillomavirus particle formation is favored, and its putative role in *in vivo* infections, will need to be characterized in future studies.

## MATERIALS AND METHODS

### Cell lines, plasmids, reagents, and viruses

HEK293TT cells were a kind gift of J. T. Schiller (NIH, Bethesda, USA) (34). HeLa cells were from ATCC. The plasmids pClneo-EGFP, p16Shell, p5LLw, and p8fwB were a kind gift from C. Buck (NIH, Bethesda, USA) (35, 64, 89). Cells were maintained in Dulbecco’s Modified Eagle’s Medium (DMEM) supplemented with 10% fetal bovine serum (FBS) and 400 µg/mL Hygromycin B in the case of HEK293TT.

DMEM, BSA standard, and SYBR Green master mix were from Thermo Fisher Scientific. OptiPrep (Iodixanol), proteinase K, heparinase, chondroitinase, and phalloidin (TRITC) were from Sigma-Aldrich. Coomassie Quick Stain was from Serva. FBS was from Biochrom, OptiMEM from Gibco, and AF488 and AF647 succinimidyl esters, as well as Lipofectamine 2000, were from Invitrogen.

### Virus preparation

HPV PsV were prepared as previously described (35). Briefly, HEK293TT cells were transfected with p16SheLL/pClneo-EGFP and p5LLw/p8fwB or p5LLw/pClneo-EGFP to produce HPV16 and HPV5 PsVs, respectively. Two days post-transfection, cells were harvested and lysed in the presence of DNase and benzonase, before the virus particles were matured for 24 h. Viruses were purified on a linear OptiPrep gradient (309,600 × *g*, 5 h) using a SW60Ti rotor (Beckman Coulter). VLP purification occurred accordingly, omitting the reporter plasmids pClneo-EGFP and p8fwB. For the “ripcord” preparation (67), DNase and Benzonase were omitted in favor of RNase to allow sedimentation of particles harboring genomic DNA loops during centrifugation.

### Enrichment of spherical HPV5 particles

To enrich spherical HPV5 from HPV5 mixtures obtained by Optiprep gradient centrifugation (band 1), these samples were subjected to velocity centrifugation on a KBr step gradient (72) (1× 50% saturated [sat.], KBr, 1× 90% sat. KBr, 2× 95% sat. KBr, and 2× sat. KBr in 10 mM Tris, pH 7.4). After the addition of samples on top of the gradient, they were subjected to 76,115 × *g* for 3 h at 20°C. The two emerging bands were collected individually and dialyzed twice against virion buffer (1× phosphate-buffered saline [PBS], 625 mM NaCl, 0.9 mM CaCl_2_, 0.5 mM MgCl_2_, 2.1 mM KCl, pH 7.4) in Float-A-Lyzer tubes (1 mL, Spectra/Por). Virus concentration was determined by densitometry using bovine serum albumin (BSA) as standard.

### Determination of viral genome equivalents (VGE)

The DNA was released from particles by incubation with 0.2 mg/mL proteinase K in HIRT buffer (400 mM NaCl, 10 mM Tris-HCl pH 7.4, 10 mM EDTA, pH 8.0) containing 0.5% SDS for 2 h at 37°C. Afterward, the DNA was purified using a Nucleo Spin Gel and PCR Clean-up kit (Macherey-Nagel) and eluted in 20 µL TE buffer (10 mM Tris, 1 mM EDTA, pH 7.5). Quantitative PCR (ABI7500, Applied Biosystems) with respective reporter plasmids as standards was used to determine the number of VGE, which was then related to L1 content of the input.

### Infection assays

About 1 × 10^5^ HeLa cells were seeded 16 h prior to infection, at which point HPV16 and HPV5 PsVs were added to the cells. After incubation for 2 h at 37°C, the inoculum was replaced by DMEM (10% FCS)/well. Cells were fixed 48 h p.i. using 4% paraformaldehyde in PBS. Cells were analyzed by flow cytometry for reporter gene expression (CytoFLEX S, Beckman Coulter).

Heparin inhibition was tested by mixing the glycan at the indicated concentrations with virus and subsequent incubation at RT for 1 h prior to infection as above. For the decoy experiments, VLPs were added in the indicated amount, with or without heparin, as described in the figure legends.

### Removal of cell surface glycosaminoglycans

Prior to infection, cells were incubated with 1 U heparinase I and III (H3917, Sigma) or chondroitinase ABC (C3667, Sigma) in 300 µL digestion buffer (10 mM HEPES, pH 7.4, 150 mM NaCl, 4 mM CaCl_2_, 0.1% BSA, 20 µg/mL cycloheximide) for 1 h at 37°C. Next, the enzyme solution was removed and replaced by 300 µL DMEM (+10% FCS) supplemented with 20 µg/mL cycloheximide and the respective PsVs.

### Electron microscopy of HPV particles and quantification

1 x 10^9^ HPV PsVs in PBS (+0.8 M NaCl) were absorbed on Formvar-coated, carbon-sputtered grids for 10 min. Next, particles were contrasted with 1% phosphotungstic acid for 7 min. Immediately after drying, samples were analyzed at 80 kV on a FEI-Tecnai 12 electron microscope (FEI, Eindhoven, Netherlands). An Olympus Veleta 4k charge-coupled device (CCD) camera was used to acquire images of selected areas. Quantification of the EM images was conducted manually by counting particles and categorizing them as spherical or filamentous.

### Electron microscopy analysis of HPV5 warts

Warts from the left shank of a patient with polymerase delta deficiency were isolated by shaving, fixed in Karnowsky’s solution (4% PFA, 1% glutaraldehyde in cacodylate buffer, pH 7.2) and tested positive for the presence of HPV5 genomic DNA, but not other common β HPV types by PCR. The warts were washed three times with cacodylate buffer (pH 7.2), post-fixed, and contrasted in 1% osmium tetroxide for 1 h at RT, followed by washing three times for 20 min in water (2× RT, 1× 4°C). Samples were then block-stained with 0.5% uranyl acetate at 4°C in the dark overnight. The sample was dehydrated the following day with ethanol in a stepwise fashion (50%, 70%, 90%, 96%, and 2× 100% ethanol; 30 min each at RT) followed by two 10-minute incubations in propylene oxide. Next, it was incubated in a solution of 50% Epon and 50% propylene oxide for 1 h, transferred to 100% Epon for 3 h at RT, and finally embedded in 100% Epon and hardened for three days. Sixty-nanometer slices were cut with a microtome (Leica-UC6 Ultramicrotome, Vienna, Austria) and counterstained with uranyl acetate and Reynold’s lead citrate for 20 min (in 25% ethanol). Finally, the slices were incubated in Reynold’s lead citrate for 2 min. The sample was imaged with the EM setup described above.

### Virus affinity measurement

8.7 µg of HPV16 and HPV5 were subjected to affinity chromatography on HiTrap Heparin HP (1 mL, VWR) on a NGC Quest System (BioRAD). Buffer A was a 10 mM sodium phosphate buffer (pH 7.4), and buffer B contained an additional 2 M NaCl. Samples were applied in 15% buffer B and washed for 30 min (1 mL/min), before being eluted with a continuous 15–75% buffer B gradient (0.5 mL/min, 10 min)

### Virus labeling

Fluorescently labeled virus was prepared according to reference 90. In brief, the respective virus preparation was mixed with AF488 or AF647 at a 1:20 molar L1:dye ratio and incubated while rotating in the dark for 1 h at RT. Afterward, the sample was centrifuged on an OptiPrep step gradient (15%, 25%, and 39%) at 225,884 × *g* for 2 h at 16°C. The emerging band was collected, supplemented with 4% glycerol, and snap-frozen in LN.

### Virus binding

As indicated, AF488- or AF647-labeled HPV preparations were incubated with or without heparin in PBS for 1 h at RT. Subsequently, the inoculum was added to 1 × 10^5^ HeLa seeds (ATCC) seeded 16 h prior to experimentation and incubated at 37°C for 1 h. Cells were washed three times with PBS and fixed in 4% PFA (PBS). Cells were stained with phalloidin-TRITC (Sigma-Aldrich) after permeabilization with 0.1% Triton X-100 in PBS. Cells were imaged using a Zeiss Axio Observer Z1 spinning disc microscope (Visitron Systems GmbH) equipped with a Yokogawa CSU22 spinning disc module and a Prime BSI camera (Photometrics). At least 10 fields of view were imaged for each condition in each of three independent experiments. The number of bound virus particles was determined for maximum-intensity projections of confocal stacks (ImageJ, version: 2.0.0-rc-68/15.2e) (91) using a CellProfiler (version: 3.1.9) (92, 93) pipeline. The number of virus particles was normalized to the cell area based on the phalloidin staining and categorized into low- and high-intensity particles according to spot sizes using manual thresholding.

## Data Availability

The complete set of original images that have been used for quantification will be available on request in a browsable, annotated form as well as for download. These large data sets have no appropriate searchable repository that meets the information standards required to reanalyze the data. Numerical data derived from these data sets will be included in the downloadable files.

## References

[1] Wang X, Huang X, Zhang Y. 2018. Involvement of human papillomaviruses in cervical cancer. Front Microbiol 9:2896. doi:10.3389/fmicb.2018.02896 30546351 PMC6279876

[2] de Martel C, Plummer M, Vignat J, Franceschi S. 2017. Worldwide burden of cancer attributable to HPV by site, country and HPV type. Int J Cancer 141:664–670. doi:10.1002/ijc.30716 28369882 PMC5520228

[3] Baker TS, Newcomb WW, Olson NH, Cowsert LM, Olson C, Brown JC. 1991. Structures of bovine and human papillomaviruses. Analysis by cryoelectron microscopy and three-dimensional image reconstruction. Biophys J 60:1445–1456. doi:10.1016/S0006-3495(91)82181-6 1663794 PMC1260204

[4] Finch JT, Klug A. 1965. The structure of viruses of the papilloma-polyoma type 3. Structure of rabbit papilloma virus, with an appendix on the topography of contrast in negative-staining for electron-microscopy. J Mol Biol 13:1–12. doi:10.1016/s0022-2836(65)80075-4 4159383

[5] KLUG A, FINCH JT. 1965. STructure of viruses of the papilloma-polyoma type. I. Human wart virus. J Mol Biol 11:403–423. doi:10.1016/s0022-2836(65)80066-3 14290353

[6] Kirnbauer R, Booy F, Cheng N, Lowy DR, Schiller JT. 1992. Papillomavirus L1 major capsid protein self-assembles into virus-like particles that are highly immunogenic. Proc Natl Acad Sci USA 89:12180–12184. doi:10.1073/pnas.89.24.12180 1334560 PMC50722

[7] Buck C.B, Cheng N, Thompson CD, Lowy DR, Steven AC, Schiller JT, Trus BL. 2008. Arrangement of L2 within the papillomavirus capsid. J Virol 82:5190–5197. doi:10.1128/JVI.02726-07 18367526 PMC2395198

[8] Buck Christopher B, Thompson CD, Pang Y-YS, Lowy DR, Schiller JT. 2005. Maturation of papillomavirus capsids. J Virol 79:2839–2846. doi:10.1128/JVI.79.5.2839-2846.2005 15709003 PMC548454

[9] Cardone G, Moyer AL, Cheng N, Thompson CD, Dvoretzky I, Lowy DR, Schiller JT, Steven AC, Buck CB, Trus BL. 2014. Maturation of the human papillomavirus 16 capsid. mBio 5:e01104-14. doi:10.1128/mBio.01104-14 25096873 PMC4128349

[10] Wolf M, Garcea RL, Grigorieff N, Harrison SC. 2010. Subunit interactions in bovine papillomavirus. Proc Natl Acad Sci USA 107:6298–6303. doi:10.1073/pnas.0914604107 20308582 PMC2852008

[11] Bzhalava D, Eklund C, Dillner J. 2015. International standardization and classification of human papillomavirus types. Virology (Auckl) 476:341–344. doi:10.1016/j.virol.2014.12.028 25577151

[12] de Villiers E-M, Fauquet C, Broker TR, Bernard H-U, zur Hausen H. 2004. Classification of papillomaviruses. Virology (Auckl) 324:17–27. doi:10.1016/j.virol.2004.03.033 15183049

[13] Mühr LSA, Eklund C, Dillner J. 2018. Towards quality and order in human papillomavirus research. Virology (Auckl) 519:74–76. doi:10.1016/j.virol.2018.04.003 29679790

[14] Bouvard V, Baan R, Straif K, Grosse Y, Secretan B, El Ghissassi F, Benbrahim-Tallaa L, Guha N, Freeman C, Galichet L, Cogliano V, WHO International Agency for Research on Cancer Monograph Working Group. 2009. A review of human carcinogens--part B: biological agents. Lancet Oncol 10:321–322. doi:10.1016/s1470-2045(09)70096-8 19350698

[15] Halec G, Schmitt M, Dondog B, Sharkhuu E, Wentzensen N, Gheit T, Tommasino M, Kommoss F, Bosch FX, Franceschi S, Clifford G, Gissmann L, Pawlita M. 2013. Biological activity of probable/possible high-risk human papillomavirus types in cervical cancer. Int J Cancer 132:63–71. doi:10.1002/ijc.27605 22514107

[16] Muñoz N, Bosch FX, Castellsagué X, Díaz M, de Sanjose S, Hammouda D, Shah KV, Meijer CJLM. 2004. Against which human papillomavirus types shall we vaccinate and screen? The international perspective. Int J Cancer 111:278–285. doi:10.1002/ijc.20244 15197783

[17] Altamura G, Tommasino M, Borzacchiello G. 2020. Cutaneous vs. Mucosal tropism: the papillomavirus paradigm comes to an “and”. Front Microbiol 11:588663. doi:10.3389/fmicb.2020.588663 33162966 PMC7591498

[18] Antonsson A, Forslund O, Ekberg H, Sterner G, Hansson BG. 2000. The ubiquity and impressive genomic diversity of human skin papillomaviruses suggest a commensalic nature of these viruses. J Virol 74:11636–11641. doi:10.1128/jvi.74.24.11636-11641.2000 11090162 PMC112445

[19] Bavinck JNB, Neale RE, Abeni D, Euvrard S, Green AC, Harwood CA, de Koning MNC, Naldi L, Nindl I, Pawlita M, Pfister H, Proby CM, Quint WGV, ter Schegget J, Waterboer T, Weissenborn S, Feltkamp MCW. 2010. Multicenter study of the association between betapapillomavirus infection and cutaneous squamous cell carcinoma. Cancer Res 70:9777–9786. doi:10.1158/0008-5472.CAN-10-0352 21098702

[20] de Koning MNC, Weissenborn SJ, Abeni D, Bouwes Bavinck JN, Euvrard S, Green AC, Harwood CA, Naldi L, Neale R, Nindl I, Proby CM, Quint WGV, Sampogna F, Ter Schegget J, Struijk L, Wieland U, Pfister HJ, Feltkamp MCW 2009. Prevalence and associated factors of betapapillomavirus infections in individuals without cutaneous squamous cell carcinoma. J Gen Virol 90:1611–1621. doi:10.1099/vir.0.010017-0 19321753

[21] Accardi R, Gheit T. 2014. Cutaneous HPV and skin cancer. Presse Med 43:e435–e443. doi:10.1016/j.lpm.2014.08.008 25451638

[22] Gheit T. 2019. Mucosal and cutaneous human papillomavirus infections and cancer biology. Front Oncol 9:355. doi:10.3389/fonc.2019.00355 31134154 PMC6517478

[23] Orth G, Jablonska S, Favre M, Croissant O, Jarzabek-Chorzelska M, Rzesa G. 1978. Characterization of two types of human papillomaviruses in lesions of epidermodysplasia verruciformis. Proc Natl Acad Sci USA 75:1537–1541. doi:10.1073/pnas.75.3.1537 206906 PMC411508

[24] Münger K, Basile JR, Duensing S, Eichten A, Gonzalez SL, Grace M, Zacny VL. 2001. Biological activities and molecular targets of the human papillomavirus E7 oncoprotein. Oncogene 20:7888–7898. doi:10.1038/sj.onc.1204860 11753671

[25] Narisawa-Saito M, Kiyono T. 2007. Basic mechanisms of high-risk human papillomavirus-induced carcinogenesis: roles of E6 and E7 proteins. Cancer Sci 98:1505–1511. doi:10.1111/j.1349-7006.2007.00546.x 17645777 PMC11158331

[26] Egawa K. 2003. Do human papillomaviruses target epidermal stem cells? Dermatology 207:251–254. doi:10.1159/000073085 14571065

[27] Schmitt A, Rochat A, Zeltner R, Borenstein L, Barrandon Y, Wettstein FO, Iftner T. 1996. The primary target cells of the high-risk cottontail rabbit papillomavirus colocalize with hair follicle stem cells. J Virol 70:1912–1922. doi:10.1128/JVI.70.3.1912-1922.1996 8627717 PMC190020

[28] Bedell MA, Hudson JB, Golub TR, Turyk ME, Hosken M, Wilbanks GD, Laimins LA. 1991. Amplification of human papillomavirus genomes in vitro is dependent on epithelial differentiation. J Virol 65:2254–2260. doi:10.1128/JVI.65.5.2254-2260.1991 1850010 PMC240574

[29] Doorbar J, Egawa N, Griffin H, Kranjec C, Murakami I. 2015. Human papillomavirus molecular biology and disease association. Rev Med Virol 25 Suppl 1:2–23. doi:10.1002/rmv.1822 25752814 PMC5024016

[30] Grassmann K, Rapp B, Maschek H, Petry KU, Iftner T. 1996. Identification of a differentiation-inducible promoter in the E7 open reading frame of human papillomavirus type 16 (HPV-16) in raft cultures of a new cell line containing high copy numbers of episomal HPV-16 DNA. J Virol 70:2339–2349. doi:10.1128/JVI.70.4.2339-2349.1996 8642661 PMC190076

[31] Klumpp DJ, Laimins LA. 1999. Differentiation-induced changes in promoter usage for transcripts encoding the human papillomavirus type 31 replication protein E1. Virology (Auckl) 257:239–246. doi:10.1006/viro.1999.9636 10208937

[32] Meyers C, Frattini MG, Hudson JB, Laimins LA. 1992. Biosynthesis of human papillomavirus from a continuous cell line upon epithelial differentiation. Science 257:971–973. doi:10.1126/science.1323879 1323879

[33] Peh WL, Middleton K, Christensen N, Nicholls P, Egawa K, Sotlar K, Brandsma J, Percival A, Lewis J, Liu WJ, Doorbar J. 2002. Life cycle heterogeneity in animal models of human papillomavirus-associated disease. J Virol 76:10401–10416. doi:10.1128/jvi.76.20.10401-10416.2002 12239317 PMC136551

[34] Buck CB, Pastrana DV, Lowy DR, Schiller JT. 2004. Efficient intracellular assembly of papillomaviral vectors. J Virol 78:751–757. doi:10.1128/jvi.78.2.751-757.2004 14694107 PMC368835

[35] Buck CB, Thompson CD. 2007. Production of papillomavirus‐based gene transfer vectors. CP Cell Biology 37:1. doi:10.1002/0471143030.cb2601s37 18228512

[36] Combita AL, Touzé A, Bousarghin L, Sizaret PY, Muñoz N, Coursaget P. 2001. Gene transfer using human papillomavirus pseudovirions varies according to virus genotype and requires cell surface heparan sulfate. FEMS Microbiol Lett 204:183–188. doi:10.1111/j.1574-6968.2001.tb10883.x 11682199

[37] Giroglou T, Florin L, Schäfer F, Streeck RE, Sapp M. 2001. Human papillomavirus infection requires cell surface heparan sulfate. J Virol 75:1565–1570. doi:10.1128/JVI.75.3.1565-1570.2001 11152531 PMC114064

[38] Johnson KM, Kines RC, Roberts JN, Lowy DR, Schiller JT, Day PM. 2009. Role of heparan sulfate in attachment to and infection of the murine female genital tract by human papillomavirus. J Virol 83:2067–2074. doi:10.1128/JVI.02190-08 19073722 PMC2643729

[39] Joyce JG, Tung JS, Przysiecki CT, Cook JC, Lehman ED, Sands JA, Jansen KU, Keller PM. 1999. The L1 major capsid protein of human papillomavirus type 11 recombinant virus-like particles interacts with heparin and cell-surface glycosaminoglycans on human keratinocytes. J Biol Chem 274:5810–5822. doi:10.1074/jbc.274.9.5810 10026203

[40] Dasgupta J, Bienkowska-Haba M, Ortega ME, Patel HD, Bodevin S, Spillmann D, Bishop B, Sapp M, Chen XS. 2011. Structural basis of oligosaccharide receptor recognition by human papillomavirus. J Biol Chem 286:2617–2624. doi:10.1074/jbc.M110.160184 21115492 PMC3024757

[41] Richards KF, Bienkowska-Haba M, Dasgupta J, Chen XS, Sapp M. 2013. Multiple heparan sulfate binding site engagements are required for the infectious entry of human papillomavirus type 16. J Virol 87:11426–11437. doi:10.1128/JVI.01721-13 23966387 PMC3807331

[42] Cerqueira Carla, Liu Y, Kühling L, Chai W, Hafezi W, van Kuppevelt TH, Kühn JE, Feizi T, Schelhaas M. 2013. Heparin increases the infectivity of human papillomavirus type 16 independent of cell surface proteoglycans and induces L1 epitope exposure. Cell Microbiol 15:1818–1836. doi:10.1111/cmi.12150 23601855 PMC4731924

[43] Feng Y, van Bodegraven D, Kádek A, L. B. Munguira I, Soria-Martinez L, Nentwich S, Saha S, Chardon F, Kavan D, Uetrecht C, Schelhaas M, Roos WH. 2024. Glycan-induced structural activation softens the human papillomavirus capsid for entry through reduction of intercapsomere flexibility. Nat Commun 15:10076. doi:10.1038/s41467-024-54373-0 39572555 PMC11582657

[44] Bano F, Soria-Martinez L, van Bodegraven D, Thorsteinsson" K, Brown AM, Fels I, Snyder NL, Bally M, Schelhaas M. 2024. Site-specific sulfations regulate the physicochemical properties of papillomavirus-heparan sulfate interactions for entry. Sci Adv 10:eado8540. doi:10.1126/sciadv.ado8540 39365863 PMC11451526

[45] Cerqueira C, Samperio Ventayol P, Vogeley C, Schelhaas M. 2015. Kallikrein-8 proteolytically processes human papillomaviruses in the extracellular space to facilitate entry into host cells. J Virol 89:7038–7052. doi:10.1128/JVI.00234-15 25926655 PMC4473586

[46] Bienkowska-Haba M, Patel HD, Sapp M. 2009. Target cell cyclophilins facilitate human papillomavirus type 16 infection. PLoS Pathog 5:e1000524. doi:10.1371/journal.ppat.1000524 19629175 PMC2709439

[47] Bronnimann MP, Calton CM, Chiquette SF, Li S, Lu M, Chapman JA, Bratton KN, Schlegel AM, Campos SK. 2016. Furin cleavage of L2 during papillomavirus infection: minimal dependence on cyclophilins. J Virol 90:6224–6234. doi:10.1128/JVI.00038-16 27122588 PMC4936150

[48] Becker M, Greune L, Schmidt MA, Schelhaas M. 2018. Extracellular conformational changes in the capsid of human papillomaviruses contribute to asynchronous uptake into host cells. J Virol 92:e02106-17. doi:10.1128/JVI.02106-17 29593032 PMC5952151

[49] Day PM, Gambhira R, Roden RBS, Lowy DR, Schiller JT. 2008. Mechanisms of human papillomavirus type 16 neutralization by l2 cross-neutralizing and l1 type-specific antibodies. J Virol 82:4638–4646. doi:10.1128/JVI.00143-08 18305047 PMC2293042

[50] Richards RM, Lowy DR, Schiller JT, Day PM. 2006. Cleavage of the papillomavirus minor capsid protein, L2, at a furin consensus site is necessary for infection. Proc Natl Acad Sci USA 103:1522–1527. doi:10.1073/pnas.0508815103 16432208 PMC1360554

[51] Selinka HC, Florin L, Patel HD, Freitag K, Schmidtke M, Makarov VA, Sapp M. 2007. Inhibition of transfer to secondary receptors by heparan sulfate-binding drug or antibody induces noninfectious uptake of human papillomavirus. J Virol 81:10970–10980. doi:10.1128/JVI.00998-07 17686860 PMC2045555

[52] Raff AB, Woodham AW, Raff LM, Skeate JG, Yan L, Da Silva DM, Schelhaas M, Kast WM. 2013. The evolving field of human papillomavirus receptor research: a review of binding and entry. J Virol 87:6062–6072. doi:10.1128/JVI.00330-13 23536685 PMC3648114

[53] Schelhaas M, Shah B, Holzer M, Blattmann P, Kühling L, Day PM, Schiller JT, Helenius A. 2012. Entry of human papillomavirus type 16 by actin-dependent, clathrin- and lipid raft-independent endocytosis. PLoS Pathog 8:e1002657. doi:10.1371/journal.ppat.1002657 22536154 PMC3334892

[54] Spoden G, Kühling L, Cordes N, Frenzel B, Sapp M, Boller K, Florin L, Schelhaas M. 2013. Human papillomavirus types 16, 18, and 31 share similar endocytic requirements for entry. J Virol 87:7765–7773. doi:10.1128/JVI.00370-13 23616662 PMC3700296

[55] Brinkert P, Krebs L, Samperio Ventayol P, Greune L, Bannach C, Amakiri C, Bucher D, Kollasser J, Dersch P, Boulant S, Stradal TEB, Schelhaas M. 2025. The actin nucleation promoting factor WASH facilitates clathrin-independent endocytosis of human papillomaviruses. EMBO Rep 26:5533–5566. doi:10.1038/s44319-025-00594-3 41073792 PMC12635285

[56] Day PM, Thompson CD, Schowalter RM, Lowy DR, Schiller JT. 2013. Identification of a role for the trans -golgi network in human papillomavirus 16 pseudovirus infection. J Virol 87:3862–3870. doi:10.1128/JVI.03222-12 23345514 PMC3624235

[57] Lipovsky A, Popa A, Pimienta G, Wyler M, Bhan A, Kuruvilla L, Guie M-A, Poffenberger AC, Nelson CDS, Atwood WJ, DiMaio D. 2013. Genome-wide siRNA screen identifies the retromer as a cellular entry factor for human papillomavirus. Proc Natl Acad Sci USA 110:7452–7457. doi:10.1073/pnas.1302164110 23569269 PMC3645514

[58] Aydin I, Villalonga-Planells R, Greune L, Bronnimann MP, Calton CM, Becker M, Lai KY, Campos SK, Schmidt MA, Schelhaas M. 2017. A central region in the minor capsid protein of papillomaviruses facilitates viral genome tethering and membrane penetration for mitotic nuclear entry. PLoS Pathog 13:e1006308. doi:10.1371/journal.ppat.1006308 28464022 PMC5412989

[59] Aydin Inci, Weber S, Snijder B, Samperio Ventayol P, Kühbacher A, Becker M, Day PM, Schiller JT, Kann M, Pelkmans L, Helenius A, Schelhaas M. 2014. Large scale RNAi reveals the requirement of nuclear envelope breakdown for nuclear import of human papillomaviruses. PLoS Pathog 10:e1004162. doi:10.1371/journal.ppat.1004162 24874089 PMC4038628

[60] Pyeon D, Pearce SM, Lank SM, Ahlquist P, Lambert PF. 2009. Establishment of human papillomavirus infection requires cell cycle progression. PLoS Pathog 5:e1000318. doi:10.1371/journal.ppat.1000318 19247434 PMC2642596

[61] Kwak K, Jiang R, Wang JW, Jagu S, Kirnbauer R, Roden RBS. 2014. Impact of inhibitors and L2 antibodies upon the infectivity of diverse alpha and beta human papillomavirus types. PLoS One 9:e97232. doi:10.1371/journal.pone.0097232 24816794 PMC4016295

[62] Broniarczyk J, Pim D, Massimi P, Bergant M, Goździcka-Józefiak A, Crump C, Banks L. 2017. The VPS4 component of the ESCRT machinery plays an essential role in HPV infectious entry and capsid disassembly. Sci Rep 7:45159. doi:10.1038/srep45159 28349933 PMC5368633

[63] Inoue T, Zhang P, Zhang W, Goodner-Bingham K, Dupzyk A, DiMaio D, Tsai B. 2018. γ-Secretase promotes membrane insertion of the human papillomavirus L2 capsid protein during virus infection. J Cell Biol 217:3545–3559. doi:10.1083/jcb.201804171 30006461 PMC6168257

[64] Buck CB, Thompson CD, Roberts JN, Müller M, Lowy DR, Schiller JT. 2006. Carrageenan is a potent inhibitor of papillomavirus infection. PLoS Pathog 2:e69. doi:10.1371/journal.ppat.0020069 16839203 PMC1500806

[65] Mistry N, Wibom C, Evander M. 2008. Cutaneous and mucosal human papillomaviruses differ in net surface charge, potential impact on tropism. Virol J 5:118. doi:10.1186/1743-422X-5-118 18854037 PMC2571092

[66] Handisurya A, Day PM, Thompson CD, Buck CB, Kwak K, Roden RBS, Lowy DR, Schiller JT. 2012. Murine skin and vaginal mucosa are similarly susceptible to infection by pseudovirions of different papillomavirus classifications and species. Virology (Auckl) 433:385–394. doi:10.1016/j.virol.2012.08.035 PMC3552393 22985477

[67] Porter SS, McBride AA. 2020. Human papillomavirus quasivirus production and infection of primary human keratinocytes. Curr Protoc Microbiol 57:e101. doi:10.1002/cpmc.101 32378811 PMC7263449

[68] Aaskov J, Buzacott K, Thu HM, Lowry K, Holmes EC. 2006. Long-term transmission of defective RNA viruses in humans and Aedes mosquitoes. Science 311:236–238. doi:10.1126/science.1115030 16410525

[69] Huang AS, Baltimore D. 1970. Defective viral particles and viral disease processes. Nature 226:325–327. doi:10.1038/226325a0 5439728

[70] Rezelj VV, Levi LI, Vignuzzi M. 2018. The defective component of viral populations. Curr Opin Virol 33:74–80. doi:10.1016/j.coviro.2018.07.014 30099321

[71] Preben von M. 1954. Incomplete forms of influenza virus. In Advances in Virus research10.1016/s0065-3527(08)60529-1 13228257

[72] Mattern CF, Takemoto KK, DeLeva AM. 1967. Electron microscopic observations on multiple polyoma virus-related particles. Virology (Auckl) 32:378–392. doi:10.1016/0042-6822(67)90288-7 4291303

[73] BREEDIS C, BERWICK L, ANDERSON TF. 1962. Fractionation of shope papilloma virus in cesium chloride density gradients. Virology (Auckl) 17:84–94. doi:10.1016/0042-6822(62)90084-3 13872737

[74] WILLIAMS RC, KASS SJ, KNIGHT CA. 1960. Structure of shope papilloma virus particles. Virology (Auckl) 12:48–58. doi:10.1016/0042-6822(60)90148-3 13855388

[75] Strobl J, Huber B, Heredia RJ, Kirnbauer R, Boztug K, Stary G. 2023. Polymerase-δ-deficiency as a novel cause of inborn cancer predisposition associated with human papillomavirus infection. Br J Dermatol 188:684–685. doi:10.1093/bjd/ljad021 36787285

[76] Doorbar J, Quint W, Banks L, Bravo IG, Stoler M, Broker TR, Stanley MA. 2012. The biology and life-cycle of human papillomaviruses. Vaccine (Auckl) 30:F55–F70. doi:10.1016/j.vaccine.2012.06.083 23199966

[77] Giachetti C, Holland JJ. 1988. Altered replicase specificity is responsible for resistance to defective interfering particle interference of an Sdi- mutant of vesicular stomatitis virus. J Virol 62:3614–3621. doi:10.1128/JVI.62.10.3614-3621.1988 2843664 PMC253502

[78] Saira K, Lin X, DePasse JV, Halpin R, Twaddle A, Stockwell T, Angus B, Cozzi-Lepri A, Delfino M, Dugan V, Dwyer DE, Freiberg M, Horban A, Losso M, Lynfield R, Wentworth DN, Holmes EC, Davey R, Wentworth DE, Ghedin E, Group IFS, Group IFS. 2013. Sequence analysis of in vivo defective interfering-like RNA of influenza A H1N1 pandemic virus. J Virol 87:8064–8074. doi:10.1128/JVI.00240-13 23678180 PMC3700204

[79] Kupke SY, Riedel D, Frensing T, Zmora P, Reichl U. 2019. A novel type of influenza A virus-derived defective interfering particle with nucleotide substitutions in its genome. J Virol 93:e01786-18. doi:10.1128/JVI.01786-18 30463972 PMC6364022

[80] Frensing T, Pflugmacher A, Bachmann M, Peschel B, Reichl U. 2014. Impact of defective interfering particles on virus replication and antiviral host response in cell culture-based influenza vaccine production. Appl Microbiol Biotechnol 98:8999–9008. doi:10.1007/s00253-014-5933-y 25132064

[81] Meng B, Bentley K, Marriott AC, Scott PD, Dimmock NJ, Easton AJ. 2017. Unexpected complexity in the interference activity of a cloned influenza defective interfering RNA. Virol J 14:138. doi:10.1186/s12985-017-0805-6 28738877 PMC5525295

[82] Frensing T, Heldt FS, Pflugmacher A, Behrendt I, Jordan I, Flockerzi D, Genzel Y, Reichl U. 2013. Continuous influenza virus production in cell culture shows a periodic accumulation of defective interfering particles. PLoS One 8:e72288. doi:10.1371/journal.pone.0072288 24039749 PMC3764112

[83] Xu J, Sun Y, Li Y, Ruthel G, Weiss SR, Raj A, Beiting D, López CB. 2017. Replication defective viral genomes exploit a cellular pro-survival mechanism to establish paramyxovirus persistence. Nat Commun 8:799. doi:10.1038/s41467-017-00909-6 28986577 PMC5630589

[84] Yoon SW, Lee SY, Won SY, Park SH, Park SY, Jeong YS. 2006. Characterization of homologous defective interfering RNA during persistent infection of vero cells with Japanese encephalitis virus. Mol Cells 21:112–120. doi:10.1016/s1016-8478(23)12908-6 16511353

[85] Sailaja G, Watts RM, Bernard HU. 1999. Many different papillomaviruses have low transcriptional activity in spite of strong epithelial specific enhancers. J Gen Virol 80 (Pt 7):1715–1724. doi:10.1099/0022-1317-80-7-1715 10423140

[86] Flint SJ, Racaniello VR, Rall GF, Hatziioannou T, Skalka AM. 2020 Principles of virology. 5th ed. Wiley & American Society for Microbiology, Hoboken, NJ.

[87] Hagan MF, Chandler D. 2006. Dynamic pathways for viral capsid assembly. Biophys J 91:42–54. doi:10.1529/biophysj.105.076851 16565055 PMC1479078

[88] Beniac DR, Melito PL, Devarennes SL, Hiebert SL, Rabb MJ, Lamboo LL, Jones SM, Booth TF. 2012. The organisation of ebola virus reveals a capacity for extensive, modular polyploidy. PLoS One 7:e29608. doi:10.1371/journal.pone.0029608 22247782 PMC3256159

[89] Buck CB, Day PM, Thompson CD, Lubkowski J, Lu W, Lowy DR, Schiller JT. 2006. Human alpha-defensins block papillomavirus infection. Proc Natl Acad Sci USA 103:1516–1521. doi:10.1073/pnas.0508033103 16432216 PMC1360544

[90] Samperio Ventayol P, Schelhaas M. 2015. Fluorescently labeled human papillomavirus pseudovirions for use in virus entry experiments. Curr Protoc Microbiol 37:14B. doi:10.1002/9780471729259.mc14b04s37 26344217

[91] Schindelin J, Arganda-Carreras I, Frise E, Kaynig V, Longair M, Pietzsch T, Preibisch S, Rueden C, Saalfeld S, Schmid B, Tinevez JY, White DJ, Hartenstein V, Eliceiri K, Tomancak P, Cardona A. 2012. Fiji: an open-source platform for biological-image analysis. Nat Methods 9:676–682. doi:10.1038/nmeth.2019 22743772 PMC3855844

[92] Carpenter AE, Jones TR, Lamprecht MR, Clarke C, Kang IH, Friman O, Guertin DA, Chang JH, Lindquist RA, Moffat J, Golland P, Sabatini DM. 2006. CellProfiler: image analysis software for identifying and quantifying cell phenotypes. Genome Biol 7:R100. doi:10.1186/gb-2006-7-10-r100 17076895 PMC1794559

[93] Kamentsky L, Jones TR, Fraser A, Bray MA, Logan DJ, Madden KL, Ljosa V, Rueden C, Eliceiri KW, Carpenter AE. 2011. Improved structure, function and compatibility for CellProfiler: modular high-throughput image analysis software. Bioinformatics 27:1179–1180. doi:10.1093/bioinformatics/btr095 21349861 PMC3072555

